# Suspected Rabies in Humans and Animals, Laikipia County, Kenya

**DOI:** 10.3201/eid2203.151118

**Published:** 2016-03

**Authors:** Mark Obonyo, James M. Akoko, Austine B. Orinde, Eric Osoro, Waqo Gufu Boru, Ian Njeru, Eric M. Fèvre

**Affiliations:** Ministry of Agriculture, Livestock and Fisheries, Nairobi, Kenya (M. Obonyo, A.B. Orinde);; Field Epidemiology and Laboratory Training Program, Nairobi (M. Obonyo, W.G. Boru);; International Livestock Research Institute, Nairobi (J.M. Akoko, E.M. Fèvre);; Kenya Zoonotic Disease Unit, Nairobi (A.B. Orinde, E. Osoro);; Ministry of Health, Kenya (W.G. Boru, I. Njeru);; University of Liverpool Institute of Infection and Global Health, Liverpool, UK (E.M. Fèvre)

**Keywords:** Rabies, zoonoses, viruses, cost, vaccine, vaccination, post-exposure prophylaxis, PEP, Kenya

**To the Editor:** Dog bites are a serious public health problem because of the associated risk for rabies virus exposure in countries to which the virus is endemic (*1*,*2*). Human rabies can be prevented by administration of postexposure prophylaxis (PEP). However, PEP rabies vaccine may be unavailable or prohibitively expensive (*3*). Delay in or failure to receive PEP after possible rabies virus exposure contributes to increased incidence of human rabies deaths (*3*).

We performed a retrospective investigation of animal bites and postbite treatment in Laikipia North sub-county, Kenya, during January 2013–February 2014. Laikipia North is 1 of 3 sub-counties in Laikipia County and has a population of 32,726 (*4*). Our investigation was instigated by 3 suspected human rabies deaths that were informally reported to the Kenya Government Zoonotic Disease Unit (ZDU) during early 2014. We reviewed animal bite records from sub-county health facilities and veterinary offices and administered a structured household questionnaire to determine outcomes, knowledge of rabies, bite management, healthcare-seeking behavior, and economic costs. This public health response was government coordinated and approved; no personal identifiers were retained.

During January 1, 2013–February 10, 2014, a total of 106 bites were recorded by 6 government-run health facilities in Laikipia North. Median reported bite incidence per month was 24 bites/100,000 persons (range 6–45 bites/100,000 persons). The median age of bite victims was 13 years (range 1–81 years); 61 (58%) bites occurred in males. Of all bites recorded, 94 (88%) were by dogs, 8 (8%) by scorpions, and 4 (4%) by humans.

The deaths of 3 humans reported to the ZDU occurred in November and December 2013. To assess whether these cases were part of an exposure cluster, we followed up on bite cases during November 1–December 31, 2013. During this period, 17 additional animal bite cases were recorded. Of these 20 bite cases, we successfully traced the households of 11 (55%) case-patients, including 2 of the 3 who died from rabies. Bites were predominantly received from owned pets (82%), and most bites (82%) were reported to be unprovoked. All bites were inflicted on extremities, and almost all (91%) were single-bite injuries (Table).

**Table:** Responses to questionnaire interview of 11 animal bite victims assessed for rabies, Laikipia County, Kenya, 2014*

Variables/categories	No. (%) case-patients
Time of bite	
Evening	6 (55)
Morning	4 (36)
Afternoon	1 (9)
Part of body bitten	
Legs	8 (73)
Arms	3 (27)
Circumstances of bite	
Unprovoked	9 (82)
Animal provoked	2 (18)
Type of animal	
Dog	10 (91)
Cat	1 (9)
Ownership of biting animal	
Owned	9 (82)
Stray	2 (18)
Rabies vaccination history of biting animal	
Unknown	7 (64)
Not vaccinated	4 (36)
Outcome of biting animal	
Alive and normal	7 (64)
Deceased	4 (36)
Wound washed after bite	
Yes	9 (82)
No	2 (18)
Treatment at healthcare facility	
Anti-tetanus	9 (82)
PEP rabies vaccination	8 (72)
Pain killers	5 (46)
Distance from nearest PEP facility, km	
>10	7 (64)
5–10	3 (27)
0–5	1 (9)
Source of PEP	
Government facility	5 (63)
Chemist	2 (25)
Private hospital	1 (13)
Costs of PEP, US$†	
No. doses of PEP administered	23
Cost categories	Average (range)
Cost/dose of PEP	≈8 (2–15)
Total cost of PEP doses	≈23 (8–50)
Direct medical cost	≈65 (2–500)
Indirect medical cost	≈34 (4–100)
Average cost for obtaining 1 dose of PEP	≈45 (8–120)
*PEP, postexposure prophylaxis. †Average annual exchange rate during 2013 was 1 Kenya shilling/$0.011586 US.	

Of 11 animals that bit case-patients, 7 had unknown histories of rabies vaccination and 4 were not vaccinated (Table). Four of the 11 animals were suspected to be rabid, including 1 cat and 3 dogs. All the suspected rabid animals were reported to exhibit aggressive or abnormal behavior, drooling or salivation, vocalization, and roaming tendencies (*5*; Technical Appendix Table 1). Three of the animals reportedly died; status was unknown for 1.

Of the 11 traced bite case-patients, 9 washed their wound before going to a healthcare facility and 8 were prescribed PEP. The median time from bite to reporting to a health facility was 1 day (range 0–3 days). Four respondents delayed in starting PEP: 3 after 3 days, and 1 after 2 days. Reasons given for delay included the high cost of PEP by 3 (including 1 who died); a health facility being too far away by 1, who died; and vaccine unavailable at nearest health facility by 2, 1 of whom died. Of 8 respondents who received PEP, 7 traveled >10 km to reach the nearest health facility. PEP availability was inconsistent at the sub-county hospital and local dispensaries; 6 of 8 respondents seeking PEP visited multiple facilities to receive PEP, including a county referral facility that was >100 km away. The World Health Organization’s 5-dose PEP regimen is recommended in Kenya (*1*). However, only 3 case-patients were prescribed and received 5 doses. Five respondents were prescribed 3, 4, or 6 doses (Technical Appendix Table 2). This finding indicates large inconsistencies in the PEP prescribing practices in this region of Kenya, a pattern that is similar in other parts of East Africa (*6*).

Respondents bore all medical costs without subsidy. Direct medical costs were ≈$2–$500 (US) per bite victim, and indirect medical costs were ≈$4–$100. The average cost of obtaining a single dose of PEP ranged from $8 to $120 (Table; Technical Appendix Table 2).

All respondents had heard of rabies. Nine (82%) knew it was transmitted to humans through a bite from a rabid dog, and 4 (36%) knew that rabies among dogs could be prevented through vaccination.

During 2014, at least 3 suspected human rabies deaths and 4 domestic animal deaths were associated with this cluster. Postbite care, including PEP, is a heavy economic burden on this community, moreso because rabies vaccine is not always locally accessible. Dog vaccination rates are low in this region and rabies in suspected animals is rarely definitively diagnosed, increasing risks for human rabies virus exposures and the economic burden of PEP administration. We recommend implementation of regular and comprehensive mass dog vaccination campaigns, in line with Kenya’s National Rabies Elimination Strategy (*7*), and further detailed studies on the epidemiology of rabies in this ecosystem, which supports human, wildlife, and domestic dog populations.

**Technical Appendix.** Questionnaire for persons bitten by animals to assess rabies status of the animals and discussion of direct and indirect costs related to rabies post-exposure prophylaxis.
