# Supplementary material for: Suspected Rabies in Humans and Animals, Laikipia County, Kenya
Source: Emerg Infect Dis. 2016 Mar;22(3):551–3. doi: 10.3201/eid2203.151118 (PMC4766878; doi:10.3201/eid2203.151118)
Supplement: Supplementary file 1 — Technical Appendix. Questionnaire for persons bitten by animals to assess rabies status of the animals and discussion of direct and indirect costs related to rabies post-exposure prophylaxis. [file 15-1118-Techapp-s1.pdf]

Article DOI: http://dx.doi.org/10.3201/eid2203.151118

Suspected Rabies in Humans and Animals, Laikipia County, Kenya, 2014

Technical Appendix

Technical Appendix Table 1. Questionnaire based on 6 clinical criteria studied to assess the rabies status of 4 suspected rabid animals that bit humans, Laikipia North sub-county, Kenya, 2014

**1) Age of the dog?**<br>a) Less than 1 month ------------------------> not rabies<br>b) One month or more or not known -----> (go to 2)
**2) State of health of the dog?**<br>a) Normal (not sick) or sick more than 10 days ---> not rabies<br>b) Sick less than 10 days or not known -----> (go to 3)
**3) How did the illness evolve?**<br><br>a) Acute onset from normal health --------> not rabies<br>b) Gradual onset or not known -------------> (go to 4)
**4) How was the condition during the clinical course in last 3-5 days?**<br>a) Stable or improving (with no treatment) --> not rabies<br>b) Symptoms and signs progressing or not known --> (go to 5)
**5) Does the dog show the sign of "circling"?**<br>*(It stumbles or walks in a circle and hits its head against the wall as if blind.)*<br>a) Yes --> not rabies<br>b) No or not known --------------------------> (go to 6)
**6) Does this dog show at least 2 of the following signs or symptoms during the last week of life?**<br>a) Yes --> rabies<br>b) No or showing only 1 sign -------------> not rabies<br>1. Drooping jaw<br>2. Abnormal sound in barking<br>3. Dry drooping tongue<br>4. Licking its own urine<br>5. Abnormal licking of water<br>6. Regurgitation
**7) Altered behavior**
**8) Biting and eating abnormal**
**9) Aggression**
**10) Biting with no provocation**<br>11. Running without apparent reason<br>12. Stiffness upon running or walking<br>13. Restlessness<br>14. Appearing sleepy<br>15. Imbalance of gait

Direct and Indirect Costs of Rabies Post-exposure Prophylaxis

For this investigation, the costs recorded included the direct medical costs, i.e. cost of biological agents (in this case only rabies vaccines, since rabies immune globulin was not administered to persons in the investigation area) and the costs associated with wound care, such as antibiotics, tetanus immunizations and disinfection. The indirect costs included out-of-pocket expenses for patients, such as transport costs to and from health facilities, accommodation and subsistence cost while seeking PEP. Market prices or local fares were used to estimate travel costs. Average cost per dose was defined as the average amount of cash spent by bite victims patients and their care giver(s) in receiving a single PEP dose. Therefore only patients who

sought and successfully obtained at least one dose of PEP were included in this calculation. This was estimated by summing all cash costs spent on obtaining PEP and dividing by the total number of doses delivered.

Technical Appendix Table 2. Costs associated with treatment of bite among 11 residents of Laikipia North sub-county in US dollars, 2014

Patient	PEP prescribed	Doses of PEP prescribed	Doses of PEP received	Cost/dose of PEP	Total cost of PEP doses	Other medical costs	Total direct costs	Total indirect costs	Total costs	Average cost of 1 PEP dose
1	Yes	4	4	$2	$8	$2	$10	$53	$63	$16
2*	Yes	5	5	$10	$50	$450	$500	$100	$600	$120
3	Yes	4	4	$10	$40	$10	$50	$58	$108	$27
4	Yes	1	1	$11	$11	0	$11	$14	$25	$25
5	Yes	3	3	$15	$45	0	$45	$35	$80	$27
6	No	0	0	0	0	$14	$14	$6	$20	0
7	No	0	0	0	0	$10	$10	$10	$20	0
8	Yes	5	5	$2	$10	$2	$12	$30	$42	$8
9†	Yes	5	5	$2	$10	$90	$50	0	$50	$10
10	No	0	0	0	0	$2	$2	0	$2	0
11	Yes	6	1	$10	$10	0	$10	$4	$14	$14

*Fatal case; high direct and indirect costs due prolonged hospitalization and burial expenses.
†Fatal case.
